# Physiological role for S-nitrosylation of RyR1 in skeletal muscle function and development

**DOI:** 10.1016/j.bbrc.2024.150163

**Published:** 2024-05-23

**Authors:** Qi-An Sun, Zachary W. Grimmett, Douglas T. Hess, Lautaro G. Perez, Zhaoxia Qian, Ruchi Chaube, Nicholas M. Venetos, Bradley N. Plummer, Kenneth R. Laurita, Richard T. Premont, Jonathan S. Stamler

**Affiliations:** a Institute for Transformative Molecular Medicine and Department of Medicine, Case Western Reserve University School of Medicine and University Hospitals Cleveland Medical Center, Cleveland, OH, 44106, USA; b Department of Surgery, Duke University School of Medicine, Durham, NC, 27710, USA; c Heart and Vascular Research Center, MetroHealth Campus of Case Western Reserve University, Cleveland, OH, 44109, USA; d Harrington Discovery Institute, University Hospitals Cleveland Medical Center, Cleveland, OH, 44106, USA

**Keywords:** S-nitrosothiol, Nitric oxide, Ryanodine receptor, Muscle hypoxia, Sarcoplasmic reticulum, Excitation-contraction coupling

## Abstract

Excitation-contraction coupling in skeletal muscle myofibers depends upon Ca^2+^ release from the sarcoplasmic reticulum through the ryanodine receptor/Ca^2+^-release channel RyR1. The RyR1 contains ~100 Cys thiols of which ~30 comprise an allosteric network subject to posttranslational modification by S-nitrosylation, S-palmitoylation and S-oxidation. However, the role and function of these modifications is not understood. Although aberrant S-nitrosylation of multiple unidentified sites has been associated with dystrophic diseases, malignant hyperthermia and other myopathic syndromes, S-nitrosylation in physiological situations is reportedly specific to a single (1 of ~100) Cys in RyR1, Cys^3636^ in a manner gated by pO_2_. Using mice expressing a form of RyR1 with a Cys^3636^→Ala point mutation to prevent S-nitrosylation at this site, we showed that Cys^3636^ was the principal target of endogenous S-nitrosylation during normal muscle function. The absence of Cys^3636^ S-nitrosylation suppressed stimulus-evoked Ca^2+^ release at physiological pO_2_ (at least in part by altering the regulation of RyR1 by Ca^2+^/calmodulin), eliminated pO_2_ coupling, and diminished skeletal myocyte contractility in vitro and measures of muscle strength in vivo. Furthermore, we found that abrogation of Cys^3636^ S-nitrosylation resulted in a developmental defect reflected in diminished myofiber diameter, altered fiber subtypes, and altered expression of genes implicated in muscle development and atrophy. Thus, our findings establish a physiological role for pO_2_-coupled S-nitrosylation of RyR1 in skeletal muscle contractility and development and provide foundation for future studies of RyR1 modifications in physiology and disease.

## 1. Introduction

The ryanodine receptor/Ca^2+^-release channel RyR1 is the principal source of stimulus-induced intracellular Ca^2+^-dependent Ca^2+^ release from the sarcoplasmic reticulum that is required for excitation-contraction coupling in skeletal muscle. RyR1 is subject to in vivo modification by multiple post-translational modifications, including phosphorylation and redox-based modifications by S-nitrosylation and S-oxidation [1–4]. Aberrant (generally increased) modification of RyR1 has been reported in multiple mouse models of muscle pathophysiology [2], notably muscular dystrophies and central core diseases [5], but the functional roles of post-translational modification of RyR1 in normal physiology remain under-characterized and controversial, reflecting at least in part the lack of genetic models in which sites of modification are altered. In particular, the effects of phosphorylation on stimulus evoked Ca^2+^ release through RyR1 are influenced by uncharacterized and ill-defined modification of channel thiols [2,6].

Of the ~100 Cys thiols in RyR1, ~80 are present in reduced form [7]. Among these, ~30 Cys thiols constitute an allosteric network that is subject to modification by S-palmitoylation, S-oxidation, and S-nitrosylation [7,8]. These alternative Cys-based modifications of RyR1 are largely non-overlapping and elicit different effects on channel function [7,8]. In this regard, it is important to appreciate that the Cys-redox landscape in RyR1 is far different under physiological conditions than under pathophysiological conditions. Direct measurements and mutagenesis experiments indicate that the amount of endogenous S-nitrosothiol (SNO) in RyR1 (the extent of S-nitrosylation) is ~1 mol SNO/mol RyR1 in normal muscle [3,8] and that the remaining thiols are present mainly in reduced state [7]. By contrast, RyR1 is extensively S-oxidized and S-nitrosylated in disease states resulting in Ca^2+^ leak [2], although these sites remain largely uncharacterized [6].

In vitro analysis of cultured cells and of subcellular fractions of skeletal muscle has demonstrated that RyR1 is subject to S-nitrosylation by physiological amounts of NO (<1 μM) and that S-nitrosylation enhances RyR1 activity [3] due to modification of a single Cys residue, Cys^3635^ in rabbit RyR1 (cognate to Cys^3636^ in mouse) [4], among the ~100 Cys in RyR1 (6). Further, S-nitrosylation of this Cys residue in cell culture models is gated by muscle oxygen tension (pO_2_), such that S-nitrosylation occurs preferentially at the low pO_2_ characteristic of active, working muscle [3,4]. In vivo, S-nitrosylation-mediated activation of RyR1 by a sarcolemma-resident neuronal NO synthase (nNOS) is activity-coupled by virtue of the Ca^2+^/calmodulin-dependence of nNOS. Thus, nNOS inhibition impairs stimulus-induced muscle performance, and these effects of endogenous NO are seen only at the physiological pO_2_ (~1 % O_2_) to which the muscle is exposed in vivo, not at ambient pO_2_ (~20 % O_2_) [9]. However, because nNOS-derived NO has numerous targets in skeletal muscle, the physiological role of RyR1 S-nitrosylation in vivo has not been established.

Regulation of RyR1 activity by S-nitrosylation may also have a role in muscle development. The expression of RyR1 and localization of nNOS to the sarcolemma both occur as the SR is established, consistent with a possible developmental role for RyR1 S-nitrosylation. Skeletal muscle development comprises two phases: production of myoblasts that acquire fusion-mediated multi-nucleate morphology before differentiating into myofibers and the subsequent acquisition by myofibers of mature morphology, including elongation by way of continued myoblast fusion and increase in myofiber diameter, as well as formation of the Ca^2+^-storing sarcoplasmic reticulum (SR) [10]. This latter phase, referred to as “hypertrophic”, occurs in the context of active muscle and thus of activity coupled intracellular Ca^2+^ flux. The activity-governed mechanisms mediating myofiber maturation operate further to couple muscle phenotype to activity throughout muscle lifetime, as evidenced by disuse atrophy and exercise-induced hypertrophy. However, the role of RyR1 in this process remains unknown, in part because knockout of RyR1 results in perinatal lethality [11].

Here, we used mice engineered to express RyR1 with the Cys^3636^→Ala mutation (C3636A) to confirm that Cys^3636^ serves as the principal locus of S-nitrosylation by endogenously generated NO in mouse skeletal muscle, and that the absence of physiological S-nitrosylation of RyR1 at Cys^3636^ suppressed stimulus-evoked Ca^2+^ release and diminished muscle contractility. We showed further that abrogation of Cys^3636^ S-nitrosylation results in disruption of hypertrophic development and maintenance of skeletal muscle. Our work thus provides foundation for further studies of Cys-based modifications of RyR1 in health and disease.

## 2. Materials and methods

### 2.1. RyR1 C3636A knock-in generation

RyR1 Cys3636Ala (C3636A) knock-in mice were generated with the Cre-loxP system. A mouse RPCl.22 BAC library was screened for RyR1, and a clone (575 O 14; insert size >80 kb) was obtained. Following analysis to map restriction enzyme sites, the clone was digested with *Eco*RI to obtain a 4 kb fragment that started within exon 72 and ended after intron 76. This *Eco*RI fragment was subcloned into a pBluescript vector containing an EcoR1 site. The C3636A mutation in exon 74 was introduced with a QuikChange kit (Stratagene) along with an *Eco*RI site between exon 75 and 76 to facilitate the screening procedure. These mutations were confirmed by DNA sequencing.

A targeting cassette (see Fig. S2A) was constructed with a pOSddACN plasmid, which contained a neomycin-resistance gene for positive selection, a negative selection marker (thymidine kinase gene), and a CRE recombinase gene driven by a testis specific promoter. This cassette was integrated so that the right arm (0.9 kb) was positioned between exon 76 and 77 and the left arm (3 kb) was positioned after the remaining portion of exon 72 (Fig. S2A). ES cells (G2:TC1) were transfected with this targeting cassette by electroporation and selected with G418 and thymidine kinase/ganciclovir. Surviving colonies were screened for RyR1 C3636A by PCR followed by Southern blotting. A pL12 plasmid containing the CRE recombinase/testis promoter cassette adjacent to RyR1 intron 76 was used as a positive control for PCR screening. Clone B7 was identified by screening, PCR products following amplification of the region containing Cys^3636^ from B7 were sequenced directly, and codons encoding both Cys^3636^ and Ala^3636^ were observed. ES cells were karyotyped and injected into blastocysts from SV129, and chimeric mice produced. A male chimera was bred to a wild-type female and pups showing by PCR a targeted and properly processed loxP locus were selected and the knocked-in sequence was confirmed. Mice were backcrossed with C57BL/6J for 10 generations. Male mice were used for all assays unless specifically noted. Mouse studies were performed under a protocol approved by the Case Western Reserve University Institutional Animal Care and Use Committee (IACUC), using procedures complying with the Guide for the Care and Use of Laboratory Animals and the American Veterinary Medical Association guidelines on euthanasia.

### 2.2. Genotyping

The presence of the knock-in mutation was determined by PCR amplification of genomic DNA using FastStart Taq polymerase for 34 cycles using the following primer set: LoxF1: GCACATTTGCTCACACAGAC, LoxR1: GCAGACTCCTAAGACCCAAGC. WT and C3636A mutants were identified by bands of 180 bp and 280bp respectively (Fig. S2B). C3636A mutation was confirmed by sequencing (TGT→GCT).

### 2.3. Preparation of SR vesicles

SR vesicles were isolated as described previously [3]. Briefly, hind-limb muscles from male 10-week-old mice were ground in liquid nitrogen using mortar and pestle, then homogenized in Biospec MiniBeadBeater-16, model 607 in ice-cold 20 mM HEPES (pH 7.4), 2 mM EDTA, 0.2 mM EGTA, 0.3 M sucrose, 100 nM aprotinin, 20 μM leupeptin, 1 μM pepstatin, 0.2 mM phenylmethylsulfonyl fluoride, and 1 mM benzamidine. Lysates were centrifuged at 10,000×*g* for 20 min, and the supernatant was centrifuged further at 100,000×*g* for 1 h. The pellet was re-suspended in lysis buffer, layered atop a continuous 20–45 % sucrose gradient (without KCl), and spun at 100,000×*g* for 14 h. SR vesicle fractions were isolated and pelleted at 120,000×*g*, resuspended in 1 % NP-40 lysis buffer (50 mM Tris-HCl pH 7.4, 5 mM EDTA, 150 mM NaCl, Roche cOmplete protease inhibitor cocktail) aliquoted, and stored in liquid nitrogen. Protein concentrations were determined with the bicinchoninic acid assay.

### 2.4. SNO-RAC assay

SNO-RAC was carried out essentially as described [12]. Briefly, SR vesicles were diluted to 0.5 mg protein/ml with HENS buffer (100 mM HEPES, 1 mM EDTA, 0.1 mM neocuproine, pH 8.0) containing 2.5 % SDS and 0.1 % methyl methanethiosulfonate and incubated at 50 °C for 20 min with frequent vortexing, followed by addition of 3 vol of cold acetone and incubation at −20 °C for 20 min. The mixture was then centrifuged at 1000 g × 5 min, washed three times with 70 % acetone and resuspended in HENS buffer containing 1 % SDS; 200 μl per mg protein). The resultant solution was added to 50 μl thiopropyl-Sepharose 6B (GE Healthcare) with or without 20 mM sodium ascorbate. Following rotation in the dark for 2–4 h, the resin was washed with 4 × 1 ml HENS buffer, then 2 × 1 ml HENS buffer diluted 1:10. Captured proteins were eluted with 50 μl 10-fold diluted HENS containing 100 mM 2-mercaptoethanol for 20 min at RT followed by Western blotting.

### 2.5. Immunoprecipitation

SR vesicles were solubilized in lysis buffer (1 % Nonidet P40, 250 mM NaCl, 5 mM EDTA, 0.02 % NaN_3_, 1 mM Na_3_VO4, 50 mM NaF, 1 mM phenylmethylsulfonyl fluoride, protease inhibitor cocktail (Promega)) and incubated overnight with anti-RyR1 mouse monoclonal Ab (Thermo Scientific MA3–925). RyR1-Ab complexes were pulled down by incubation for 2 h at room temperature with Dynabeads-Protein G (Invitrogen) and analyzed by Western blotting with the precipitating anti-RyR1 Ab, anti-CaM monoclonal mouse Ab (Thermo Scientific MA3–917) or anti-FKBP12 rabbit polyclonal Ab (Thermo Scientific PA1–026A).

### 2.6. Assay of RyR1 activity by ^3^H-Ryanodine binding

RyR1 activity measured as [^3^H]-ryanodine binding was assayed as described previously [3]. For [^3^H]-ryanodine binding at high and low pO_2_, isolated SR vesicles were incubated overnight at room temperature with 5 nM [^3^H]-ryanodine in 20 mM imidazole, 125 mM KCl (pH 7.0), 0.3 mM Pefabloc (Roche), 30 μM leupeptin, and 10 μM CaCl_2_. The reactions were bubbled continuously with 1 % or 20 % O_2_ containing 5 % CO_2_ plus N_2_. Nonspecific binding was defined using 5 μM cold ryanodine. In specified samples, SR vesicles were preincubated at the two O_2_ tensions with 1 μM CaMBP (to remove CaM from RyR1). After incubation, samples were diluted with 20 vol of ice-cold H_2_O and filtered through Whatman GF/B filters presoaked with 2 % (wt/wt) polyethyleneimine. After washing filters with 5 mL buffer (1 mM Pipes, 0.1 M KCl, pH 7.0) three times, filter counts were quantified in a liquid scintillation counter.

### 2.7. Intact muscle bioassay

Muscle bioassays were performed as described previously [13]. EDL muscles were from 10-week-old male mice, and both EDL muscles from two mice were assessed in parallel in 4 baths. Each muscle was strung from the tendons and hung on a force transducer in an organ bath superfused with Kreb’s solution (pH 7.4) at 37 °C. Muscles were equilibrated for 10 min at 20 % O_2_/5 % CO_2_/remainder N_2_, after which the first test protocol was performed at 20 % O_2_ then switched to 1 % O_2_ (both in 5 % CO_2_/remainder N_2_) for the second test. Muscle tension was adjusted to maximal isometric twitch-force generation (optimal muscle length, L_o_). The stimulation protocol used trains of square-wave pulses (train duration 75 ms; individual pulse duration, 2 ms; pulse frequency, 40–250 Hz; pulse amplitude, 60 V, which is about 75 % of the amplitude that elicited maximal twitch force) from submerged platinum electrodes (7.0 mm wide) parallel to the long axis of the muscle. There was at least 2 min rest between trains of tetanic stimuli. Fatigue resistance was assessed at 20 % O_2_ by delivering continuous tetanic stimulation at 100 Hz and measuring the interval over which tension diminished from maximal to half-maximal. For each muscle, L_o_ and the wet weight of the muscle were measured at the end of testing; the cross-sectional area was calculated assuming the muscle was a cylinder of length L_o_ with 1.06 g cm^−3^ density [13]. Force generation was normalized to EDL cross-sectional area (N/cm^2^).

### 2.8. Assay of intracellular Ca^2+^ release in primary myocytes

Single flexor digitorum brevis hind-limb muscle fibers were isolated from male mice and assayed as described previously [13]. Dissected muscle bundles were incubated in Tyrode’s solution containing 2 mg/mL collagenase (type II; Worthington) for 3 h at 37 °C, then triturated in DMEM with BSA and 50 U/mL penicillin, 50 mg/mL streptomycin. Individual myofibers were pelleted by centrifugation at 1000×*g* for 3 min, then were incubated in complete DMEM at 37 °C under 5 % CO_2_ for 2–3 days. Myofibers were incubated with Fluo 3-AM (5 μM) for 45 min with or without PEG-catalase (10 units/mL), then suspended in Tyrode’s solution inside a sealed chamber for field stimulation (RC-21BRFS; Warner Instruments) with a continuous flow of room temperature Tyrode’s solution that had been bubbled with 1 % or 20 % O_2_ (in 5 % CO_2_ and N_2_). Myofibers imaged on an inverted Axiovert microscope (Zeiss) were simulated at 1 Hz (1 ms duration; ~40–50 V). Ca^2+^ signals, imaged as Fluo 3-AM fluorescence emission, were captured with a PentaMAX CCD camera (Princeton Instruments) at a frame rate of 217 Hz. Ca^2+^ transient amplitude was calculated as the difference between peak and baseline levels, normalized to baseline fluorescence (F/F_o_).

### 2.9. qPCR

The Skeletal Muscle Myogenesis and Myopathy RT2 Profiler PCR Array (Qiagen) was employed to assess mRNA levels associated with eighty-four genes implicated in skeletal muscle myogenesis and myopathy in EDL muscle from WT and C3636A male mice at 4 and 12 weeks of age. Cellular RNA was prepared from skeletal muscle using Trizol reagent followed by RNA purification and qPCR employed the iQ SYBR green supermix with ABI ViiA7 detection system (Bio-Rad). Data were analyzed with the Qiagen web-based RT2 profiler PCR array data analysis software.

### 2.10. Western blot analysis of protein expression

For western blotting of mouse tissue, hind limb muscle lysates were prepared in RIPA buffer (Sigma) supplemented with protease inhibitors (Roche). Lysate proteins were quantified using BCA (Bio Rad) and separated by SDS-PAGE on 4–12 % acrylamide gels (Criterion, Bio Rad, Hercules, CA) for detection with anti-target antibodies diluted in TBS-Tween with 5 % BSA at the indicated concentrations (Mstn: ab203076 at 1:200, Mapk8: ab199380 at 1:2500, Ppargc1a: ab54481 at 1:1000, Rps6kb1: ab32359 at 1:1000, and GAPDH: sc-365062 at 1:4000). For immunoblot detection, transferred nitrocellulose membranes were cut to include the size range of interest, blocked with 5 % milk in TBS-Tween, exposed sequentially to primary and HRP-conjugated secondary antisera, and detected by KwikQuant camera upon exposure to ECL reagent (Cell Signaling). Quantification of band strength was done in ImageJ, and graphing and statistical analysis were done in Prism (GraphPad), with sample values normalized to GAPDH and shown as relative quantity with WT 4-week-old sample value set as 1.

### 2.11. In vivo assessment of muscle performance

In vivo muscle function tests were carried out employing standard methods by the Rodent Behavioral Core at Case Western Reserve University with 12–20-week-old male mice. Before each test, mice underwent three training sessions at 2-day intervals and at least 1 day elapsed between test sessions. Evaluation of grip strength measured the force (g) achieved before the limb released from a receding tension bar. A hanging wire test assessed hang time until release, and mice underwent three trials per session, with a 30 s recovery period between trials. Maximal running speed was assessed with a horizontal treadmill equipped with a shocker plate [14,15]. During testing, the treadmill was operated at a given speed for 10 s, and a successful trial required that the mouse follow that speed for 3 s. Speed on succeeding trials was increased by 2 m/s. Treadmill endurance was assessed by operating the treadmill at 8.5 m/min for 10 min followed by increases of speed by 2.5 m/min at 3 m intervals until mice did not attempt to re-engage the treadmill.

### 2.12. Microscopy and image quantification

EDL muscles were harvested from male mice and immediately mounted in OCT and flash-frozen in an isopentane bath suspended in liquid nitrogen, then transferred to dry ice and sectioned with 10 μm thickness onto microscope slides, within the middle third of the muscle. Slides were blocked with 5 % BSA in PBS, stained with either no primary antibody (negative control; NC) or one primary antibody against a myosin subtype (detailed below) overnight at 4 °C. Slides were washed, stained with secondary antibodies (detailed below), washed again and cover slips were mounted with Vectashield, stored at 4 °C before imaging on a Leica DMi8 confocal microscope. The following myosin primary antibodies were obtained from the Developmental Studies Hybridoma Bank (created by the NICHD of the NIH and maintained at The University of Iowa, Department of Biology, and deposited to the DSHB by S. Schiaffino) and used at the indicated concentrations: mouse BA-F8 (anti-MYH7; slow. 1:50), mouse SC-71 (anti-MHC type 2A. 1:50) and mouse BF–F3 (anti-MHC type 2B. 1:3). Rabbit Laminin antiserum (PA1–16730; 1:500) was obtained from Invitrogen. The following secondary antibodies were used for visualization: anti-rabbit IgG (A11036 AlexaFluor 555), anti-mouse IgG (A11001 AlexaFluor 488) and anti-mouse IgM (A21042 AlexaFluor 488). All secondaries were used at 1:200. All analysis was performed in ImageJ software.

### 2.13. pO_2_ measurement in muscle

pO_2_ was measured using polarographic oxygen electrode (≈10 μm diameter glass micropipette) placed within the muscle core, and was calibrated at each use. Muscle was equilibrated for 10 min in each gas mixture, first 20 % O_2_ then 95 % O_2_ and finally 1 % O_2_. At 1 % and 20 % O_2_, muscle core oxygen tension was 3.5 mmHg and 40.7 mmHg respectively, consistent with previous results^15^.

### 2.14. Quantification and statistical analysis

**Fig. 1.**
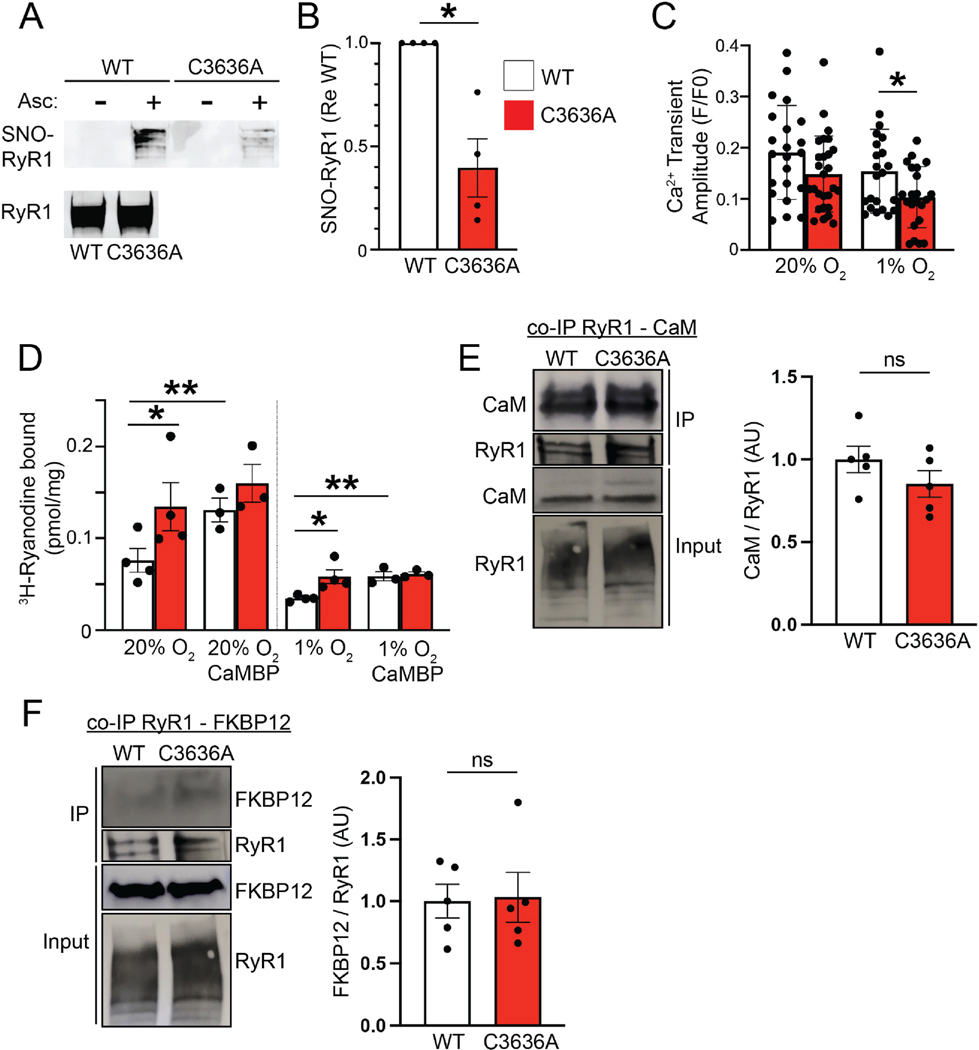
Effects of C3636A mutation on RyR1 S-nitrosylation, evoked and basal RyR1 activities, and protein-protein interactions. (**A**) Representative Western blot (upper) of SR vesicles isolated from WT or C3636A hindlimb muscles and subjected to the resin-assisted capture of S-nitrosylated proteins (SNO-RAC) method in room air. Omission of ascorbate (Asc) provides a control for SNO assay specificity. Blotting for RyR1 (lower) shows the RyR1 content of starting material. (**B**) Quantification of data in (A); n = 4; *p < 0.05. (**C**) Analysis of depolarization-evoked Ca^2+^ transients in WT or C3636A FDB myofibers at 1 % or 20 % O_2_. *p < 0.05; n = 22–27 myofibers per group. (**D**) Basal RyR1 activity in SR vesicles from C3636A or WT muscle was assessed by ^3^H-ryanodine binding at 1 % O_2_ or 20 % O_2_. 1 μM CaMBP was preincubated with SR vesicles where indicated. *p < 0.05C3636A compared to WT, **p < 0.05 CaMBP compared to untreated, †p < 0.05 20 % O_2_ compared to 1 % O_2_. (**E**, **F**) RyR1 immunoprecipitates from SR vesicles at 20 % O_2_ were immunoblotted for co-immunoprecipitated CaM (E) or FKBP12 (F), and for immunoprecipitated RyR1 (lower). Samples in (E,F) contained equal amounts of SR protein and RyR1 (Fig. S2C) and were normalized to immunoprecipitated RyR1, with representative Western blots of immunoprecipitated proteins shown; n = 4 mice per group.

Analysis of Western blot data was carried out with ImageJ (NIH), Microsoft Excel (Fig. 1) and GraphPad Prism (Fig. 4); analysis of muscle contractility was performed with SAS software (version 9.3; Cary, NC).

All values are expressed as mean ± SEM, and n represents experimental replications. Unpaired Student’s *t*-test was used for post-hoc comparison between two groups and one-way ANOVA with appropriate post-test (Tukey-Kramer) for comparison between multiple groups. The linear mixed model accounting for repeated measures [16] was used to analyze muscle contraction evoked by stimulation. Values of p < 0.05 were considered statistically significant.

## 3. Results

### 3.1. Generation of C3636A mice

Rabbit Cys^3635^ (mouse Cys^3636^) is adjacent to the Ca^2+^/calmodulin binding site in RyR1 [17] within the shell-core linker peptide between the bridge solenoid and core solenoid domains (as defined in the cryo-EM structures of the rabbit RyR1 homotetramer), at a region where the folded B- and C-solenoids also meet the folded junctional-solenoid and N-terminal domain, but at some distance from the channel pore (Fig. S1) [18,19]. To test the effect of preventing S-nitrosylation of this site, we created a mouse with knock-in mutation of Cys^3636^ to Ala (C3636A) in the *Ryr1* gene using the Cre-loxP system (Fig. S2A). Mutant mice were backcrossed with wild-type (WT) C57BL/6J for 10 generations before testing, and genotyping (Fig. S2B) and DNA sequencing confirmed the C3636A mutation. The amount of C3636A RyR1 protein in homozygote mice was indistinguishable from that of WT RyR1 in littermate controls (Fig. 1A; Fig. S2C), consistent with a report of an independent RyR1 C3636A knock-in mouse strain [20].

### 3.2. Diminished S-nitrosylation of C3636A RyR1 in vivo

We have shown that exogenously expressed rabbit RyR1 is S-nitrosylated on Cys^3635^ in HEK293 cells exposed to exogenous NO at 1 % O_2_ (characteristic of working muscle) but not at ambient pO_2_ (20 % O_2_), and that C3635A mutation eliminates the pO_2_-dependent enhancement of RyR1 activity induced by treatment with exogenous NO [4]. We therefore first assessed the effects of C3636A mutation on S-nitrosylation of mouse RyR1 in vivo. We found that S-nitrosylation of RyR1 in vivo was substantially diminished (~60 %) in skeletal muscle of C3636A mice at baseline as assessed by the SNO-RAC method (resin-assisted capture of S-nitrosylated proteins) [12] (Fig. 1A and B), demonstrating Cys^3636^ as the principal target of S-nitrosylation in situ by endogenous sources of NO. This is in agreement with a report that Cys3636 is the primary RyR1 SNO-site in mouse hippocampal slices at baseline and after NMDA receptor activation [20]. The presence of residual S-nitrosylation of C3636A RyR1 is consistent with the report of an additional site(s) of S-nitrosylation in RyR1 in SR vesicles derived from rabbit skeletal muscle exposed to NO or to S-nitroso-glutathione, or in microsomes derived from rabbit C3635A-mutant RyR1-transfected HEK 293 cells [1], potentially reflecting Cys sites with lesser propensity for modification (which may be revealed by elimination of highly reactive Cys3635/3636).

### 3.3. Cys^3636^ mutation impairs pO_2_-regulated Ca^2+^ release through RyR1

We have previously assessed intracellular Ca^2+^ flux within isolated myofibers from flexor digitorum brevis (FDB; a predominantly fast-twitch foot muscle), induced by transient depolarization and monitored with the Ca^2+^-sensitive fluorescent dye Fluo3-AM, to evaluate the role of oxidative modification in regulating Ca^2+^ release through RyR1 [21]. To assess the effect of the C3636A mutation on stimulus-induced Ca^2+^ release through RyR1, we examined the Ca^2+^ transients evoked by transient depolarization of myofibers isolated from FDB from mice exposed to 1 % O_2_ and 20 % O_2_, conditions under which stimulus-induced increases in intracellular Ca^2+^ reflect predominantly the activation of RyR1 [9]. We found that the amplitude of Ca^2+^ transients was significantly diminished in FDB myofibers from C3636A mice compared to those from WT mice at 1 % O_2_ (at which S-nitrosylation operates normally) but not at 20 % O_2_ (at which S-nitrosylation does not occur) (Fig. 1C; Fig. S3), demonstrating that S-nitrosylation of Cys^3636^ facilitates stimulus-induced Ca^2+^ release under conditions found in working muscle. Mikami et al. also reported that Ca^2+^ transients were normal in myofibers from their RyR1 C3636A mice when assessed under ambient conditions (~20 % O_2_); they did not test physiological levels of pO_2_ [20].

We next assessed the effect of C3636A mutation on RyR1 activity by quantifying ^3^H-ryanodine binding (which occurs on the active channel and therefore measures P_O_, open channel probability) in SR vesicles derived from hind-limb and axial muscle [3,9] from WT and C3636A mice. Confirming previous reports [4,7], ryanodine binding was significantly higher in 20 % O_2_ compared to 1 % O_2_ in both WT and C3636A samples (Fig. 1D), reflecting activation of RyR1 by oxidative modification at 20 % O_2_, as reported previously [3,9]. However, C3636A RyR1 activity was significantly increased compared to that of WT RyR1 at both 1 % O_2_ (~60 % enhancement) and 20 % O_2_ (~70 % enhancement) (Fig. 1D). Enhanced RyR1 P_O_ in the C3636A mutant under basal conditions would represent a “Ca^2+^ leak” from the SR, reflecting loss of regulation by CaM under basal conditions [4].

The activity of RyR1 is inhibited by Ca^2+^-bound calmodulin (CaM) [22], whereas removal of CaM from RyR1 by treatment with CaM-binding protein (CaMBP) enhances basal RyR1 activity [3]. Cys^3635/3636^ resides within the CaM binding site of RyR1 [17], and S-nitrosylation of rabbit RyR1 at Cys^3635^ inhibits CaM binding, blocking CaM repression of RyR1 activation [3]. S-nitrosylation does not inhibit the activity of RyR1 that has been stripped of CaM [3]. We therefore tested whether C3636A mutation altered RyR1 interaction with CaM or CaM-regulated RyR1 activity in vitro. We found that treatment of isolated SR vesicles with CaMBP significantly enhanced WT RyR1 activity (as measured by ^3^H-ryanodine binding) at both 1 % O_2_ and 20 % O_2_, but did not affect the ryanodine-binding activity of C3636A RyR1 under high or low oxygen conditions (Fig. 1D). To determine whether C3636A mutation affected the ability of RyR1 to bind CaM, we immunoprecipitated RyR1 from SR vesicles and examined association with CaM by co-immunoprecipitation. Consistent with previous in vitro results showing that binding of exogenous CaM to rabbit RyR1 expressed in HEK293 cells was not affected by C3635A mutation [4], CaM coimmunoprecipitated with WT and C3636A RyR1 in mouse SR vesicles to a similar extent (Fig. 1E). Thus, C3636A mutation does not appear to substantially alter association of RyR1 with CaM but disrupts CaM-dependent regulation of basal RyR1 activity in a pO_2_-independent fashion. C3636A mutation thus contributes to an active configuration, reflected in enhanced basal P_O_, as seen previously for the effect of S-nitrosylation of this Cys residue occurring selectively at low pO_2_ [4]. Cys→Ala mutation of a SNO site can mimic the effect of SNO, specifically affecting the association of function-regulating proteins, including the N-ethylmaleimide sensitive factor [23] and β–arrestin2 [24]. We propose that C3636A mutation may also mimic the effect of SNO on the interaction of RyR1 and CaM.

Altered S-nitrosylation of RyR1 has been associated with loss of FKBP12 binding to RyR1 and pathophysiological Ca^2+^ leak through RyR1 in multiple mouse models [2]. However, the abundance of FKBP12 in SR vesicles did not differ between WT and C3636A mice and the C3636A mutation did not affect FKBP12 binding to RyR1 as assessed by coimmunoprecipitation from SR vesicles (Fig. 1F).

### 3.4. Cys^3636^ mutant RyR1 refractory to S-nitrosylation exhibits impaired muscle function at physiological pO_2_

**Fig. 2.**
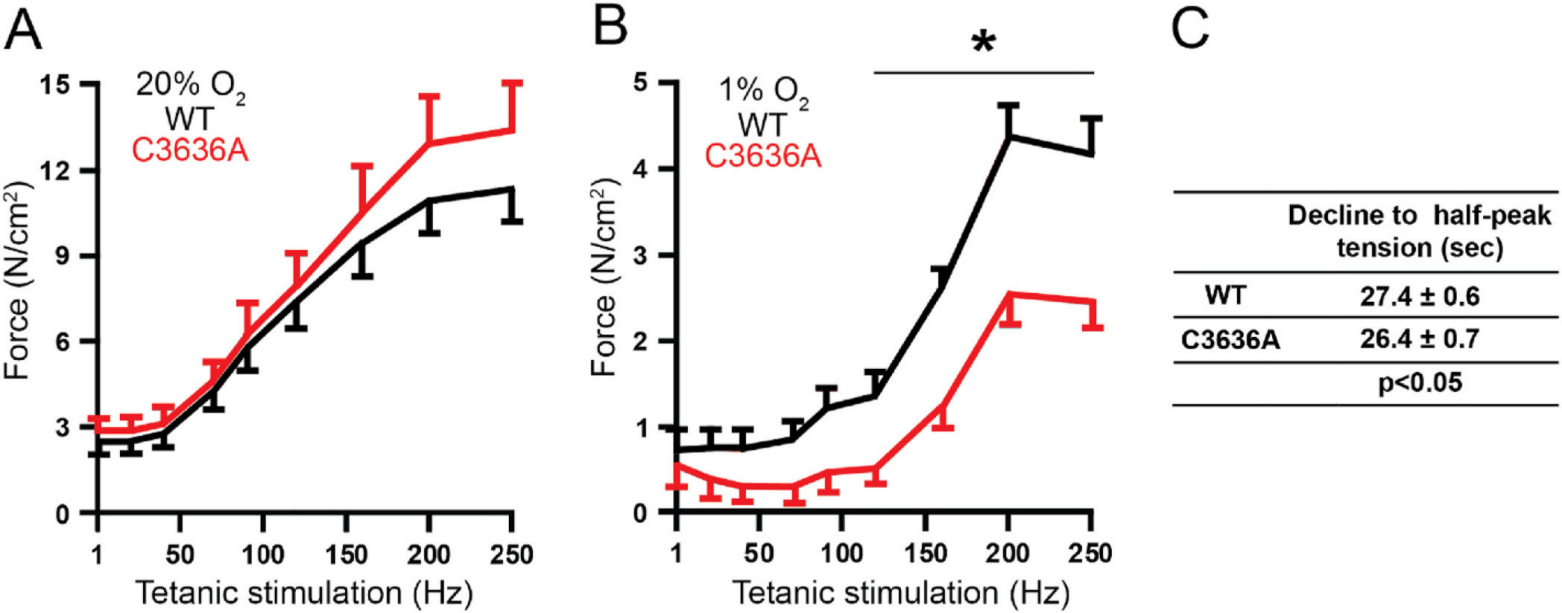
C3636A mutation results in diminished EDL muscle force production. (**A**) Tetanic force production (1–250 Hz) by intact WT (black) and C3636A (red) EDL muscle assessed in vitro at 20 % O_2_; *p < 0.05. (**B**) Tetanic force production (1–250 Hz) by intact WT and C3636A EDL muscle assessed in vitro at 1 % O_2_. *p < 0.01. (**C**) The decline from peak to half-peak tension during sustained tetanic stimulation at 100 Hz. n = 24–28 individual EDL muscles from 7 to 8 mice per genotype.

We have previously shown that the effect of NO derived from nNOS on force production in skeletal muscle is pO_2_ dependent and mediated through RyR1 [21]. To link these functional consequences with S-nitrosylation of RyR1, we assessed the effect of C3636A mutation on muscle function by assaying force production (N/cm^2^) by the predominantly fast-twitch (myosin heavy chain/MHC type 2-dominant) hind-limb muscle extensor digitorum longus (EDL), as previously used to study NO/redox regulation of RyR1 [9,21]. At 20 % O_2_, specific force production during tetanic stimulation was similar in C3636A and WT EDL (Fig. 2A, Fig. S4), indicating that the C3636A mutation did not alter contractile capacity. However, force production at 1 % O_2_, which mimics physiological muscle pO_2_ [21], was significantly lower in C3636A EDL than in WT EDL over the tetanic frequency range of 120–250 Hz (Fig. 2B). This can be understood by appreciating that the effects of S-nitrosylation are mediated via CaM, whereas CaM binding is disrupted in RyR C3636A [4]. Thus, S-nitrosylation of Cys^3636^ potentiates force production at physiological pO_2_ at which S-nitrosylation of Cys^3636^ is enabled, consistent with the previous finding that pharmacological inhibition of nNOS suppresses specific force production at 1 % O_2_ [21]. In addition, during sustained tetanic stimulation (100 Hz), the time-course of the decline from peak tension was significantly shortened in C3636A EDL compared to WT EDL, indicative of diminished acute fatigue resistance resulting from elevated basal Ca^2+^ release (Fig. 2C).

### 3.5. Aberrant development of skeletal muscle in the absence of Cys^3636^

**Fig. 3.**
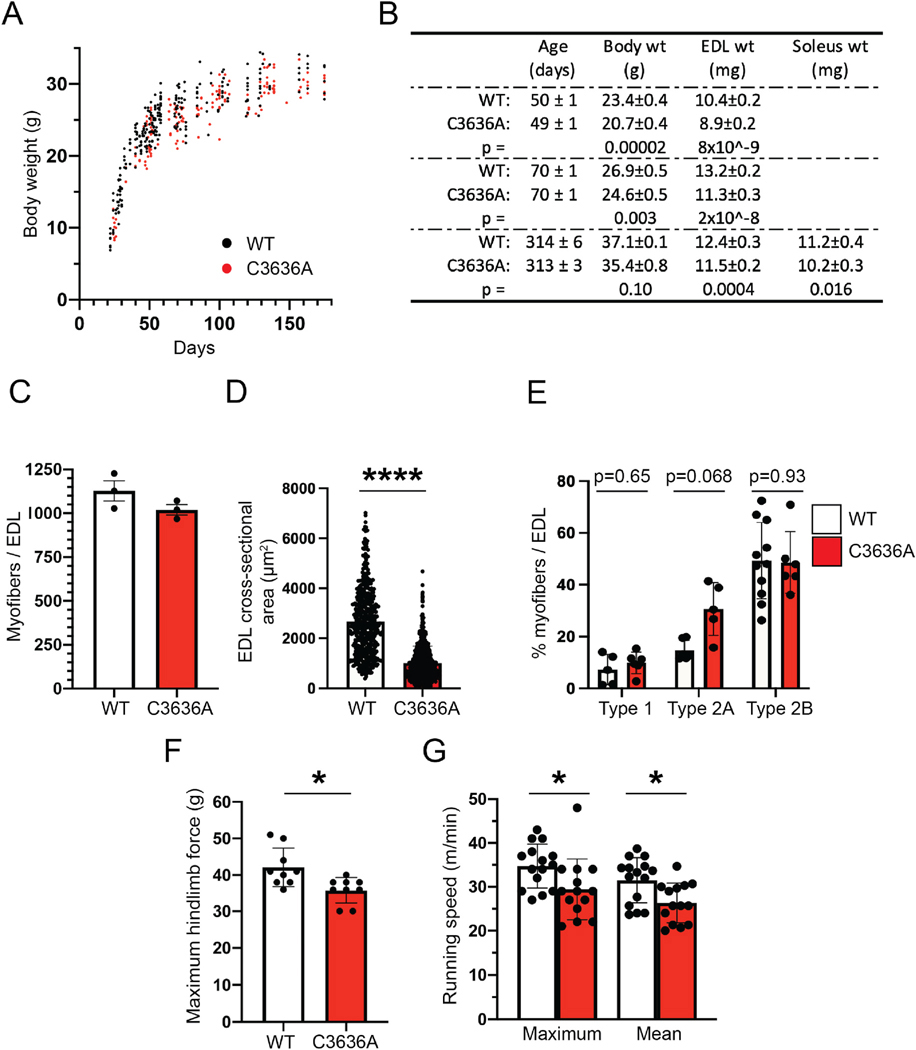
Deficient hypertrophic skeletal muscle development in RyR1 C3636A mice. (**A**) Body weight of male WT and C3636A mice at the indicated ages. Each data point represents a single mouse. (**B**) Body weight, EDL weight and soleus weight in male WT and C3636A mice. At 50 days, n = 12–16 mice per genotype; at 70 days, n = 12–14 mice per genotype; at 314 days, n = 7–11 mice per genotype. (**C** and **D**) Quantification of myofiber number (C) and myofiber cross-sectional area (D) from cross-sections of EDL muscle from WT and C3636A mice. (**E**) Quantification of myofibers with positive immunohistochemical staining for type 1, type 2A or type 2B MHC markers in EDL muscle from WT and C3636A mice. Each dot represents an individual EDL muscle cross-section. Results in (C) to (E) were obtained from 150 to 170 myofibers from at least 3 individual mice per genotype. (**F**) Maximum hind-limb grip force in C3636A mice; *p < 0.05; n = 9 mice per genotype. (**G**) Maximal and mean (3 trials) running speed in C3636A mice. *p < 0.05, **p < 0.01, ****p < 0.0001; n = 14–15 mice per genotype.

C3636A mice weighed less than WT mice from ~30 days of age until weight stabilized at ~180 days of age (Fig. 3A–B), indicating decreased muscle mass, and this difference persisted until at least ~300 days of age (Fig. 3B), with a similar phenotype observed in female C3636A mice (Fig. S5). At 50 days of age, this difference represented about a 12 % reduction in body weight. Diminished muscle mass was demonstrated directly by weighing the EDL (Fig. 3B) and the soleus, a predominantly slow-twitch (MHC type 1-dominant) hind-limb muscle (Fig. 3B). The proportional reduction in muscle mass closely matched the reduction in total body weight (Fig. 3B). EDL length was similar in 10-week-old WT and C3636A mice (Fig. 3B). In addition, there were no differences in the weights of heart, lung, liver or kidney between WT and C3636A mice (Table S1). Thus, this developmental defect in C3636A mice appears to be specific to skeletal muscle mass, with little effect on muscle length or the mass of other major tissues.

Morphometric analysis of cross sections of EDL from 90-day-old mice revealed that myofiber number was similar in C3636A and WT mice (Fig. 3C), whereas cross-sectional area was significantly decreased in C3636A mice (~62 %) (Fig. 3D). In addition, fiber-type specific staining for myosin heavy chain (MHC) isoforms 1, 2A and 2B revealed a modest increase in type 2A fibers (that was not statistically significant) and no change in type 1 or 2B fibers (Fig. 3E; Fig. S6). Overall fiber type distribution was consistent with previously published studies in murine EDL muscle (40,41). Thus, C3636A mice have significantly diminished fiber diameter with largely similar fiber type composition.

We confirmed the developmental deficiency by in vivo muscle function tests. Both hind limb grip strength (Fig. 3F) and forced maximal running speed (exercise tolerance test) (Fig. 3G) were significantly diminished in C3636A mice compared to WT mice, whereas treadmill endurance (Fig. S7A) and wire hang time (Fig. S7B) did not differ, consistent with a primary effect of C3636A mutation on force generation.

### 3.6. Molecular phenotype of C3636A mutants

**Fig. 4.**
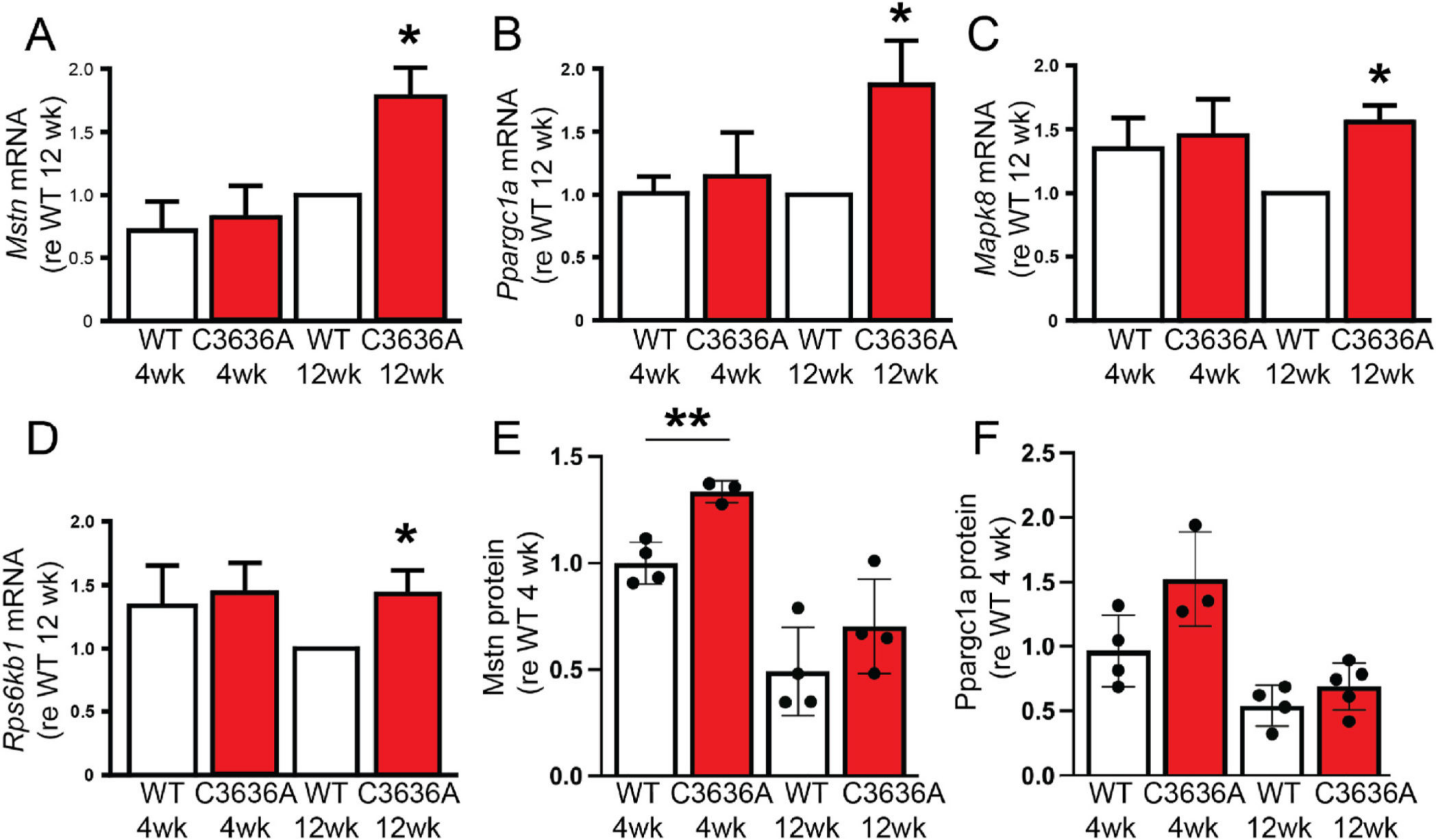
C3636A mutation results in altered expression of genes and gene products involved in myogenesis and myopathy. (**A-D**) qPCR analysis for *Mstn* (A), *Ppargc1a* (B), *Mapk8* (C) and *Rps6kb1* (D) in hind-limb muscle from WT or C3636A mice at 4 or 12 weeks of age. n = 4 mice per group; *p < 0.05 compared to WT. (**E,F**) Western blots for Mstn (E) and Ppargc1a (PGC1α) (F) in hind-limb muscle from WT or C3636A mice at 4 or 12 weeks of age. Protein expression was normalized to GAPDH. n = 3–4 mice per group; **p < 0.01.

To assess the transcriptional correlates of aberrant hypertrophic development associated with dysregulated Ca^2+^ release through C3636A RyR1, we used qPCR analysis to assess the mRNA levels of eighty-four genes implicated in skeletal muscle myogenesis and myopathy in EDL muscle. Consistent previous findings, we observed substantial changes between 4 and 12 weeks of age in the expression of transcription of markers of skeletal muscle development (Table S2). These markers can be classified broadly as associated with myogenesis, hypertrophy, atrophy, contractility, autocrine signaling or metabolism. 26 of 84 assayed transcripts showed increases or decreases in mRNA abundance of between 1.33-fold and 15-fold between 4 and 12 weeks in WT and/or C3636A mice (Table S2). No significant differences were seen between the abundance of any assayed transcript in WT and C3636A mice at 4 weeks of age (Table S2). However, at 12 weeks of age, four transcripts whose abundance showed a significant >1.33-fold increase in C3636A muscle compared to WT muscle: *Mstn*, encoding myostatin, a member of the TGF-β superfamily (Fig. 4A; Table S2); *Ppargc1a*, encoding PGC-1α (Fig. 4B; Table S2); *Mapk8*, encoding mitogen-activated protein kinase 8, also known as c-Jun N-terminal kinase (JNK1) (Fig. 4C; Table S2); *Rps6kb1*, encoding ribosomal protein S6 kinase beta-1 (Fig. 4D; Table S2). The enhanced mRNA abundances of *Mstn* and *Ppargc1a* represent up-regulation, whereas those of *Mapk8* and *Rps6kb1* represent a failure to down-regulate (Fig. 4A–D).

At the protein level, small but not statistically significant increases in Mstn and PGC-1α (Fig. 4E and F) and no difference in Mapk8 or Rps6kb1 (Figs. S8A and B) were observed in muscle from 12-week old C3636A mice. However, the protein level of Mstn was significantly higher in C3636A muscle than in WT muscle at 4 weeks of age (Fig. 4E), consistent with the well-characterized role of myostatin as a myokine that negatively regulates muscle mass [25].

## 4. Discussion

Our findings demonstrate a physiological role for S-nitrosylation of RyR1 in Ca^2+^ release and muscle contractility and suggest a mechanism for coupling RyR1 activity with muscle function. Firstly, production of NO by nNOS is coupled to intracellular Ca^2+^ levels and thus to muscle activity, and enhanced nNOS activity results in enhanced S-nitrosylation of RyR1 [26]. Secondly, muscle activity lowers tissue pO_2_, which is required for RyR1 S-nitrosylation by altering the redox state of an allosteric network of thiols [3]. In this way, muscle activity is regulated by pO_2_ in vivo. Previous findings based on pharmacological inhibition of nNOS were consistent with a contractile effect of RyR1 S-nitrosylation that is seen only at physiological pO_2_ [21], and thus masked in RyR1 assays performed in room air. The present findings directly demonstrate the facilitatory effect of RyR1 S-nitrosylation in vivo, which is localized to a single Cys residue out of ~100, Cys^3635/3636^, and which has essential roles in muscle strength and development.

Embryonic development is characterized by low pO_2_ [27], and nNOS deficiency impairs muscle development at early stages of perinatal growth [28]. Our findings support a specific role for NO-regulated Ca^2+^ release from RyR1 in the hypertrophic development and maintenance of skeletal muscle, which is impaired by C3636A mutation and is associated with a distinct gene expression signature. Thus, the facilitated Ca^2+^ release from RyR1 resulting from S-nitrosylation of Cys^3636^ evidently provides an essential signal for myofiber growth that is not adequately supplied by nNOS or RyR1 activities alone. These findings augment understanding of the central role of activity coupled intracellular Ca^2+^ release in skeletal muscle transcriptional and translational regulation that is required for normal growth [29].

In contrast to its suppressive effects on stimulus-evoked activity, C3636A mutation enhanced RyR1 basal activity (*P*_O_ as assessed by ^3^H-ryanodine binding). An increase in basal activity by Cys^3636^ mutation is consistent with our finding that removing CaM with CaMBP under basal conditions activates WT but not C3636A RyR1 and indicates disrupted regulation of mutant RyR1 by CaM. These data are also consistent with previous findings that C3636A mutation in vitro resulted in loss of CaM-dependent NO modulation [4] and that CaM-dependent activation of RyR by S-nitrosylation occurs without CaM displacement [3]. Together, our findings establish a role for Cys^3636^ in the regulation of RyR1 by CaM and demonstrate the physiological importance of S-nitrosylation of Cys^3636^ in modulating RyR1 function.

Ca^2+^ leak through RyR1 has been attributed to hyper-S-nitrosylation in several mouse models of myopathy, including those for muscular dystrophy [30], central core disease and malignant hypothermia [31], extreme exercise-induced fatigue [32], and ventilator-induced diaphragmatic dysfunction [33]. However, in all cases, S-nitrosylation is accompanied by RyR1 mutations and/or additional, aberrant post-translational modifications including oxidative modification and phosphorylation, as well as loss of FKBP12 binding (where examined). Thus, a causal relationship between aberrant RyR1 S-nitrosylation and either Ca^2+^ leak or muscle dysfunction has not been clearly established. In contrast, the enhanced basal activity of C3636A RyR1 that we observe, which can be ascribed to aberrant CaM-dependent regulation of RyR1, was not associated with disruption of FKBP12 binding. Additionally, both Ca^2+^ release through C3636A RyR1 and force production were impaired only at low pO_2_ at which S-nitrosylation of RyR1 can occur, indicating intact contractile function. Thus, the CaM-based enhancement of basal activity we observe may not contribute directly to pathophysiology. In sum, Ca^2+^ release through S-nitrosylation of Cys^3636^, which requires low pO_2_, is likely unrelated mechanistically to Ca^2+^ release mediated by multi-site S-nitrosylation and S-oxidation [8] as seen in disease, and instead operates physiologically during exercise and development.

Notably, mice with Cys^3636^ mutation recapitulate the developmental changes seen in mice lacking nNOS [28]. In both, myofiber diameter and muscle mass are diminished, thus identifying this NO-mediated effect with RyR1. Collectively, these findings are consistent with a growth-promoting role for S-nitrosylation of RyR1 at Cys^3636^ in hypertrophic development. Conversely, mice lacking S-nitrosoglutathione reductase (GSNOR), a denitrosylase that removes SNO from proteins, demonstrate RyR1 hypernitrosylation (at uncharacterized sites and amounts) with reduced muscle and body weight, but with elevated specific force and reduced fatigue [34]. S-nitrosylation-mediated release of excess Ca^2+^ (under low oxygen) may thus augment Ca^2+^-dependent transcriptional processes, altering long-term muscle function.

**Fig. 5.**
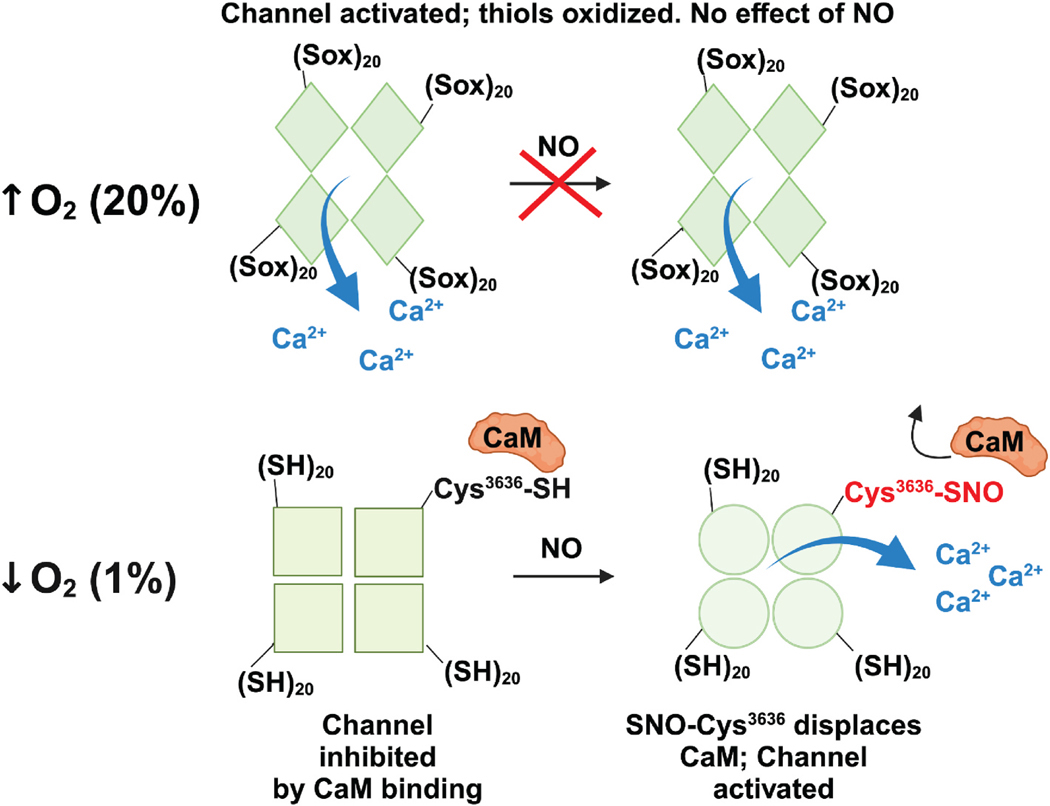
RyR1 is a hypoxia-regulated redox sensor and NO transducer. Schematic diagram of RyR1 allostery under high and low oxygenation, and the effect of S-nitrosylation at Cys^3636^ (thiol labeled as Cys^3636^-SH or as S^3636^–SNO), among an allosteric network of ~20 thiols ((SH)_20_ in diagram) that are subject to oxidation ((Sox)_20_ in diagram). S-nitrosylation of RyR1 at Cys^3636^ in low O_2_ conditions (1 % O_2_) displaces calmodulin (CaM), allowing Ca^2+^ flux through the channel. In normoxia (20 % O_2_), the allosteric thiol network comprised of ~20 Cys residues is oxidized, increasing Ca^2+^ flux but limiting NO-based regulation of RyR1 channel activity through CaM. Image created with BioRender.com.

Our findings present a nuanced picture for physiological regulation of skeletal muscle function in which RyR1 acts as both an O_2_ sensor and O_2_-regulated NO signal transducer acting through CaM (Fig. 5), thereby influencing skeletal muscle activity and development. S-nitrosylation of this single site in vivo under physiological conditions in mouse RyR1 confirms our prior mapping of this site in human RyR1 in model systems, and demonstrates that this single site accounts for much of physiological regulation of RyR1 by NO, in marked contrast with hypernitrosylation of RyR1 in disease. Ca^2+^ release through RyR1 that is regulated by S-nitrosylation at Cys^3635/3636^ thus provides an NO-dependent molecular mechanism through which activity and growth are coupled in healthy skeletal muscle.

## Supplementary Material

1

## Data Availability

All data needed to evaluate the conclusions in the paper are present in the paper or the Supplementary Materials.

## Appendix A. Supplementary data

Supplementary data to this article can be found online at https://doi.org/10.1016/j.bbrc.2024.150163.

## Abbreviations

CaM: calmodulin
CaMBP: calmodulin binding protein
EDL: extensor digitorum longus muscle
nNOS: neuronal NO synthase, product of the *Nos1* gene
pO_2_: oxygen tension
RyR1: skeletal muscle ryanodine receptor/Ca^2+^-release channel, product of the *Ryr1* gene
SNO: S-nitrosothiol
SR: muscle sarcoplasmic reticulum
