# Supplementary material for: Physiological role for S-nitrosylation of RyR1 in skeletal muscle function and development
Source: Biochem Biophys Res Commun. Author manuscript; Available in PMC 2025 Nov 26. (PMC12651786; doi:10.1016/j.bbrc.2024.150163)
Supplement: 1 [file NIHMS2119077-supplement-1.pdf]

Supplementary Materials for Sun *et al*.

Supplemental Tables S1 and S2

Supplemental Figures S1-S8

Supplemental Table S1 (related to Figure 3A,B).

Organ weights did not vary between WT and C3636A mice; n=7-11 mice per group. P value was determined by two-tailed t-test.

	Age (days)	Heart (g)	Lung (g)	Liver (g)	Kidney (g)
C3636A	312.7±3	0.19±0.01	0.21±0.01	1.66±0.07	0.25±0.01
WT	313.9±56	0.19±0.01	0.24±0.02	1.58±0.07	0.24±0.01
P value		0.81	0.11	0.40	0.064

Supplemental Table S2 (related to Figure 4).

qPCR measurement of muscle genes. Relative abundances of mRNA (via qPCR) for 84 transcripts associated with skeletal muscle myogenesis and myopathy, in WT and C3636A mice at 4 and 12 weeks of age; n=4 mice per group at each age. The p-value and associated fold-change are highlighted in red wherever p<0.05. Transcripts with >1.33-fold difference between WT and C3636A at 12 weeks of age are highlighted in blue.

	WT 12 versus 4 weeks		C3636A 12 versus 4 weeks		C3636A versus WT 4 weeks		C3636A versus WT 12 weeks	
	p value	Fold-change	p value	Fold-change	p value	Fold- change	p value	Fold- change
Acta1	0.374	-1.254	0.731	-1.090	0.931	1.078	0.384	-1.161
Actn3	0.889	-1.011	0.250	1.261	0.696	-1.074	0.497	1.846
Acvr2b	0.276	1.296	0.019	1.841	0.975	1.014	0.123	1.129
Adipoq	0.973	1.088	0.409	-1.624	0.476	1.369	0.764	1.448
Adrb2	0.681	1.426	0.335	1.848	0.783	1.135	0.255	1.108
Agrn	0.019	-2.723	0.212	-1.781	0.390	-1.558	0.861	1.194
Akt1	0.025	-1.819	0.065	-1.925	0.420	-1.140	0.768	-1.092
Akt2	0.095	1.309	0.043	1.347	0.909	1.011	0.815	1.275
Atp2a1	0.964	1.027	0.046	1.384	0.899	-1.009	0.046	-1.055
Bcl2	0.004	-1.957	0.048	-2.485	0.502	1.148	0.691	1.377
Bmp4	0.398	-1.522	0.271	-1.607	0.833	1.085	0.917	1.133
Camk2g	0.545	1.154	0.023	1.440	0.822	1.069	0.001	1.141
Capn2	0.699	-1.166	0.146	-1.430	0.364	1.321	0.995	-1.484
Capn3	0.392	-1.244	0.322	-1.219	0.940	1.028	0.892	1.231
Casp3	0.196	-2.274	0.005	-3.746	0.747	1.051	0.349	-3.822
Cast	0.960	1.019	0.409	-1.151	0.539	1.142	0.805	1.767
Cav1	0.145	-1.840	0.100	-2.254	0.583	1.186	0.829	-2.589
Cav3	0.074	-1.524	0.375	-1.596	0.478	1.104	0.658	1.293
Cryab	0.849	1.347	0.628	-1.131	0.956	1.131	0.486	1.253
Cs	0.004	-4.030	0.070	-1.915	0.509	-1.209	0.134	1.339
Ctnnb1	0.014	-1.675	0.106	-1.454	0.718	1.054	0.089	-1.235
Dag1	0.027	-1.490	0.099	-1.434	0.805	-1.051	0.943	-1.222
Des	0.450	1.122	0.600	1.085	0.756	1.019	0.917	-1.234
Dmd	0.536	-1.159	0.766	1.078	0.499	1.167	0.056	-1.013
Dmpk	0.074	-2.095	0.621	-1.385	0.554	-1.163	0.378	-1.665
Dysf	0.071	-1.671	0.369	-1.529	0.756	1.041	0.594	1.013

Fbxo32	0.002	8.622	0.000	6.269	0.162	1.410	0.941	1.521
Fgf2	0.625	-1.106	0.156	1.323	0.555	-1.115	0.193	1.521
Foxo1	0.962	1.139	0.958	1.135	0.496	1.381	0.218	1.521
Foxo3	0.200	-1.355	0.537	-1.158	0.941	1.002	0.467	1.521
Hdac5	0.358	1.201	0.182	1.714	0.552	1.075	0.270	1.534
Hk2	0.881	-1.014	0.386	1.179	0.882	1.020	0.484	1.449
Igf1	0.848	-1.380	0.092	-2.082	0.400	1.330	0.537	1.375
Igf2	0.002	-10.849	0.000	-15.079	0.062	-1.587	0.306	-2.176
Igfbp3	0.136	-2.648	0.152	-2.568	0.980	1.089	0.511	1.138
Igfbp5	0.301	-1.326	0.409	1.201	0.939	-1.031	0.141	1.566
Ikbkb	0.003	-1.716	0.081	-1.589	0.208	-1.220	0.678	-1.115
Il1b	0.355	2.079	0.560	-1.218	0.970	-1.142	0.348	-2.853
Il6	0.578	1.616	0.524	-1.053	0.809	-1.059	0.402	-1.777
Lep	0.179	-2.598	0.380	-2.271	0.520	-1.124	0.734	1.032
Lmna	0.482	-1.348	0.321	-1.563	0.763	1.037	0.873	-1.104
Mapk1	0.281	-1.329	0.582	-1.081	0.951	-1.001	0.205	1.246
Mapk14	0.568	-1.184	0.976	-1.005	0.627	-1.098	0.865	1.088
Mapk3	0.008	-1.797	0.068	-1.806	0.458	-1.246	0.139	-1.236
**Mapk8**	0.335	-1.277	0.971	1.130	0.759	1.065	**0.045**	**1.558**
Mb	0.362	-1.336	0.330	-1.599	0.548	1.722	0.298	1.458
Mef2c	0.321	1.242	0.133	1.385	0.554	-1.142	0.852	-1.011
Mmp9	0.375	1.645	0.001	1.923	0.357	-1.236	0.530	-1.043
**Mstn**	0.114	1.702	0.007	2.473	0.626	1.176	**0.014**	**1.732**
Musk	0.046	3.623	0.002	3.866	0.109	1.294	0.492	1.400
Myf5	0.724	-1.181	0.138	-2.747	0.789	-1.086	0.264	-2.493
Myf6	0.154	1.491	0.070	1.404	0.550	1.190	0.588	1.135
Myh1	0.018	-3.638	0.018	-3.719	0.542	1.229	0.503	1.219
Myh2	0.282	-1.319	0.609	1.120	0.990	1.010	0.083	1.512
Myod1	0.622	1.112	0.849	1.120	0.421	-1.338	0.376	-1.311
Myog	0.022	-3.925	0.000	-6.052	0.095	-1.320	0.258	-2.008
Myot	0.608	-1.097	0.769	-1.074	0.630	1.345	0.241	1.392
Neb	0.458	-1.188	0.934	1.061	0.817	1.165	0.136	1.488
Nfkb1	0.925	-1.018	0.581	-1.074	0.755	1.022	0.709	-1.019
Nos2	0.092	-2.671	0.157	-3.362	0.944	1.092	0.626	-1.138
Pax3	0.428	-1.403	0.794	1.424	0.793	-1.317	0.556	1.538
Pax7	0.015	-2.868	0.005	-2.688	0.629	-1.074	0.924	1.007
Pdk4	0.067	2.220	0.764	-1.087	0.197	1.914	0.262	-1.244
Pparg	0.422	-1.183	0.108	-2.012	0.746	1.465	0.462	-1.146
**Ppargc1a**	0.732	1.044	0.143	3.049	0.669	-1.582	**0.009**	**1.871**
Ppargc1b	0.021	-1.746	0.046	-1.761	0.254	1.138	0.621	1.144
Ppp3ca	0.861	1.068	0.553	1.313	0.772	1.178	0.061	1.467

Prkaa1	0.174	-1.731	0.131	-1.570	0.868	1.005	0.629	1.123
Prkab2	0.348	1.143	0.033	1.453	0.704	-1.064	0.247	1.210
Prkag1	0.968	-1.019	0.871	-1.023	0.779	-1.088	0.973	-1.078
Prkag3	0.164	-1.582	0.535	1.102	0.241	-1.368	0.263	1.292
Rhoa	0.494	-1.322	0.176	-1.450	0.910	1.041	0.642	-1.041
**Rps6kb1**	0.309	-1.242	0.816	1.005	0.714	1.103	**0.047**	**1.395**
Sgca	0.816	1.146	0.612	1.173	0.827	1.107	0.467	1.148
Slc2a4	0.805	-1.038	0.463	1.245	0.752	-1.132	0.783	1.157
Tgfb1	0.538	-1.653	0.032	-2.530	0.884	1.032	0.351	-1.464
Tnf	0.110	-4.269	0.037	-2.619	0.400	-1.325	0.802	1.247
Tnnc1	0.745	1.185	0.132	-6.217	0.292	1.928	0.287	-3.771
Tnni2	0.375	-1.246	0.830	1.334	0.941	1.063	0.104	1.791
Tnnt1	0.299	-2.024	0.139	-8.271	0.712	1.578	0.486	-2.555
Tnnt3	0.658	1.181	0.122	1.348	0.753	1.133	0.035	1.310
Trim63	0.126	1.714	0.152	1.753	0.608	1.225	0.249	1.270
Ttn	0.090	-1.341	0.638	-1.078	0.536	1.076	0.153	1.357
Utrn	0.055	-1.552	0.021	-1.997	0.805	1.042	0.163	-1.218

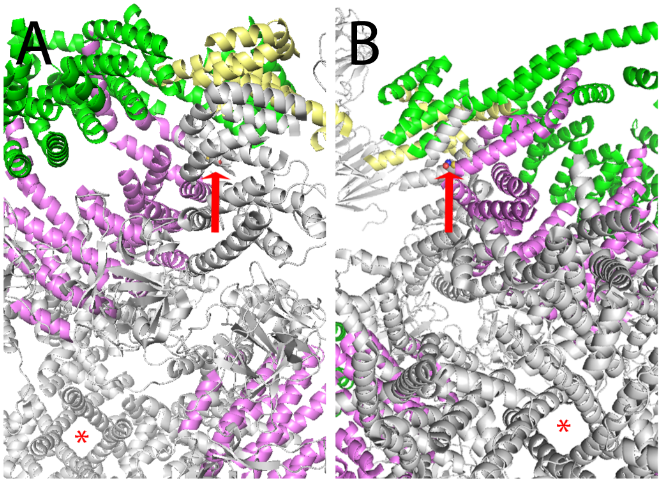

Fig. S1. SNO-Cys^{3635} in the rabbit RyR1 structure (Related to Figure 1).

(A) Extracellular domains of RyR1 looking down toward membrane.

(B) Extracellular domains of RyR1 looking up away from membrane.

Cys^{3635}-SNO (cognate to mouse Cys^{3636}) is indicated by red arrows. Red "*" indicates channel pore. Domains are color-coded green=B-solenoid, cyan=C-solenoid, yellow=J-solenoid as defined in the cryo-EM structure of rabbit RyR1 [18]. Modified from the structure of des George et al. [18] in PyMol to add SNO at Cys^{3635} and color-code domains.

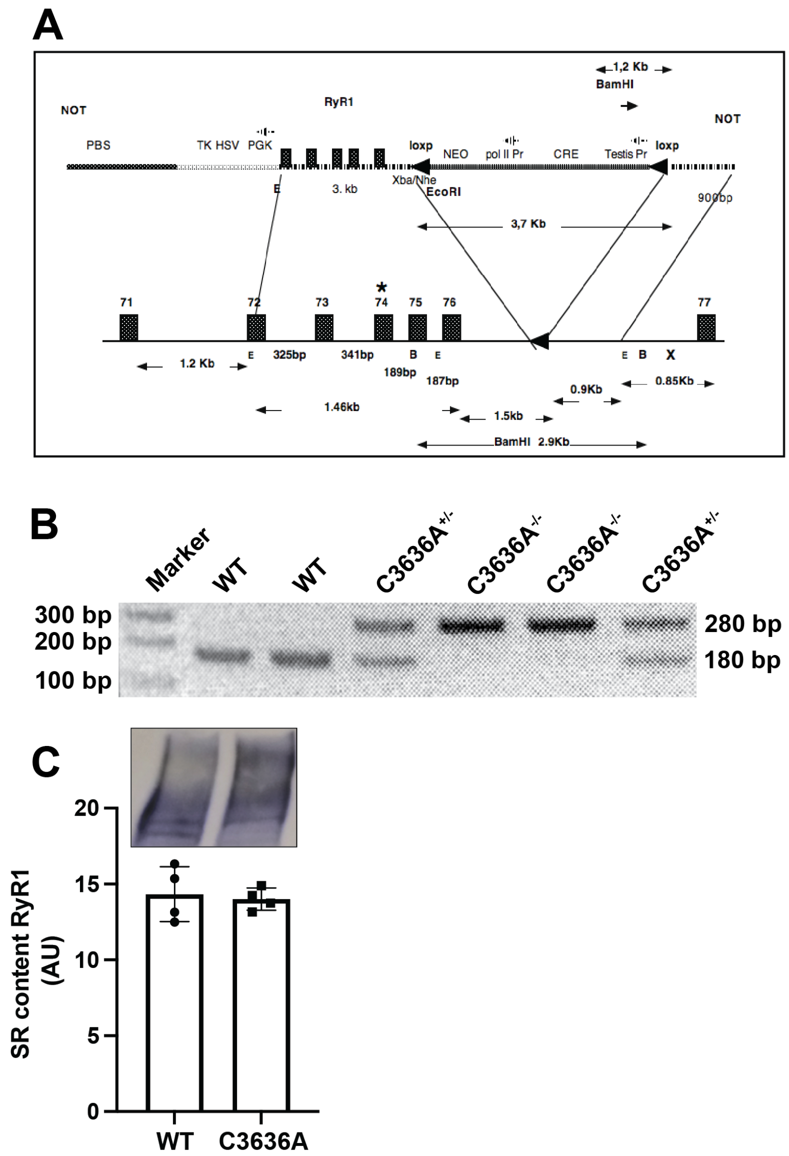

Fig S2. Creation of RyR1 C3636A mutant mice (Related to Figure 1).

(A) The targeting vector employed in the creation of RyR1 C3636A knock-in mice (top) and the corresponding numbered exons of the mouse RyR1 gene (bottom).

(B) Confirmation by genotyping of C3636A mutation.

(C) Levels of expression of WT and C3636A RyR1 did not differ as assessed by Western blotting for RyR1 in the SR vesicle subcellular fraction; n=4 blots with distinct SR preparations.

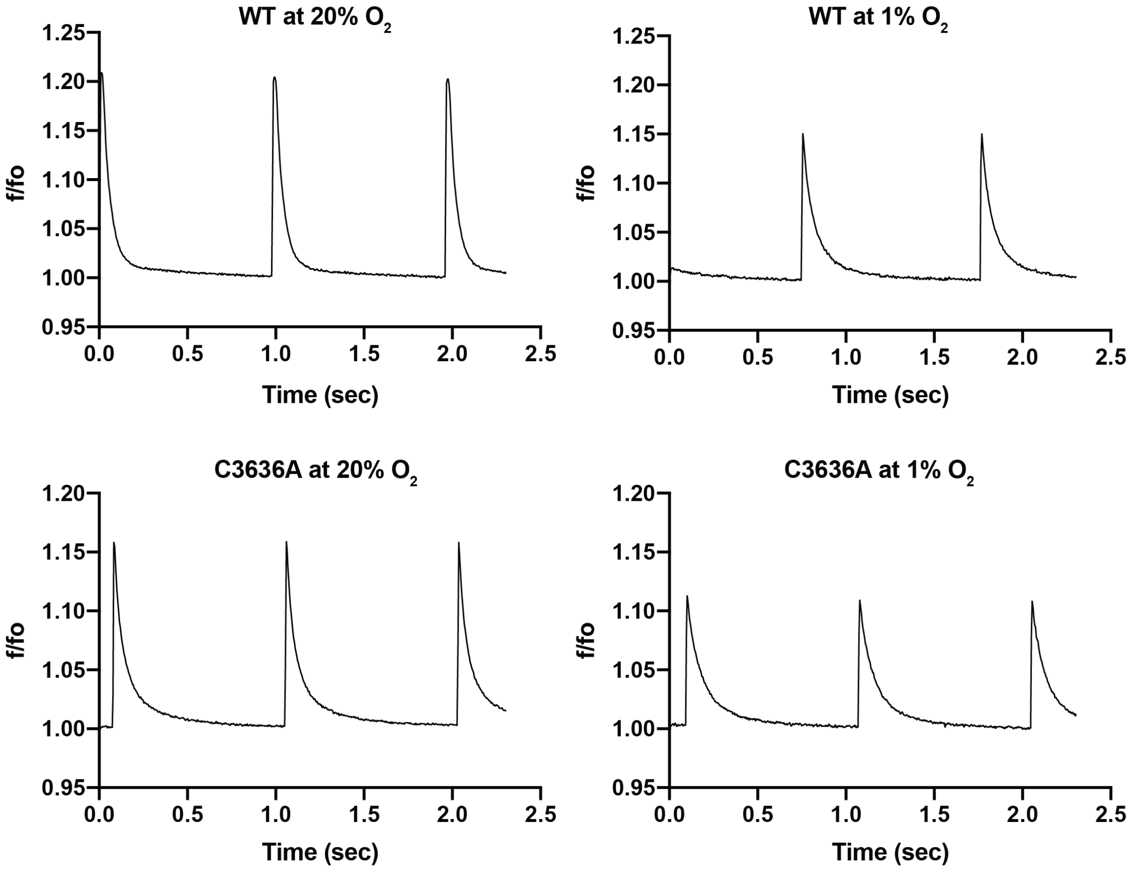

Fig S3. Exemplar calcium traces from isolated flexor digitorum brevis myofibers (Related to Figure 1). Isolated myofibers were loaded with the Fluo3-AM calcium indicator and depolarized by 1 millisecond electrical field stimulation (1 Hz). Cells from WT and C3636A mice were assessed under 20% oxygen and 1% oxygen: (A) WT cells at 20% oxygen. (B) WT cells at 1% oxygen. (C) C3636A cells at 20% oxygen. (D) C3636A cells at 1% oxygen. Traces are plotted as stimulated fluorescence over basal fluorescence (F/Fo).

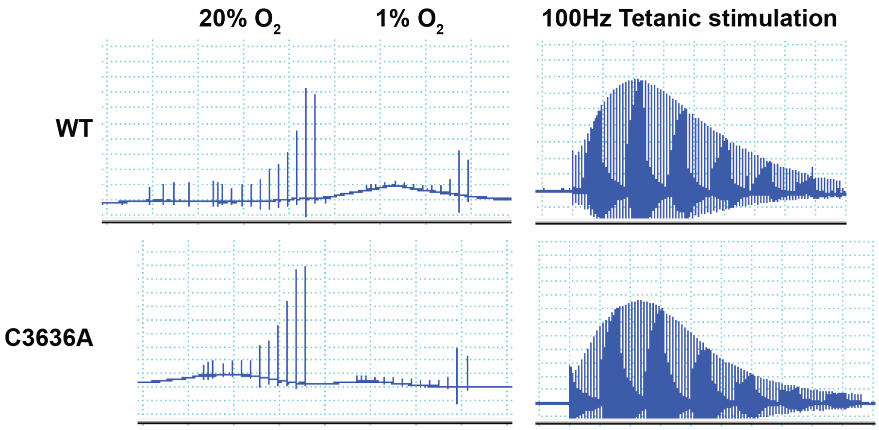

Fig. S4. Exemplar tension traces from EDL muscle organ bath assay (Related to Figure 2).
Electrically stimulated tensions were obtained for EDL muscle from WT or C3636A mice as indicated, under room air (21% O_2), then under 1% O_2, prior to tetanic stimulation under 20% O_2 to calculate half-relaxation time. Both EDL muscles were tested from each of 2 mice in each experimental run (only 1 is shown).

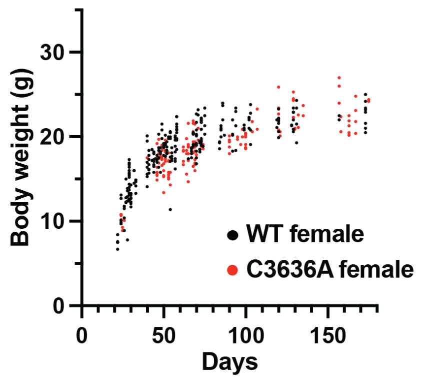

Fig. S5. Body weight of female WT and C3636A mice (Related to Figure 3). Body weight of female WT and C3636A mice at the indicated ages. Each data point represents a single mouse.

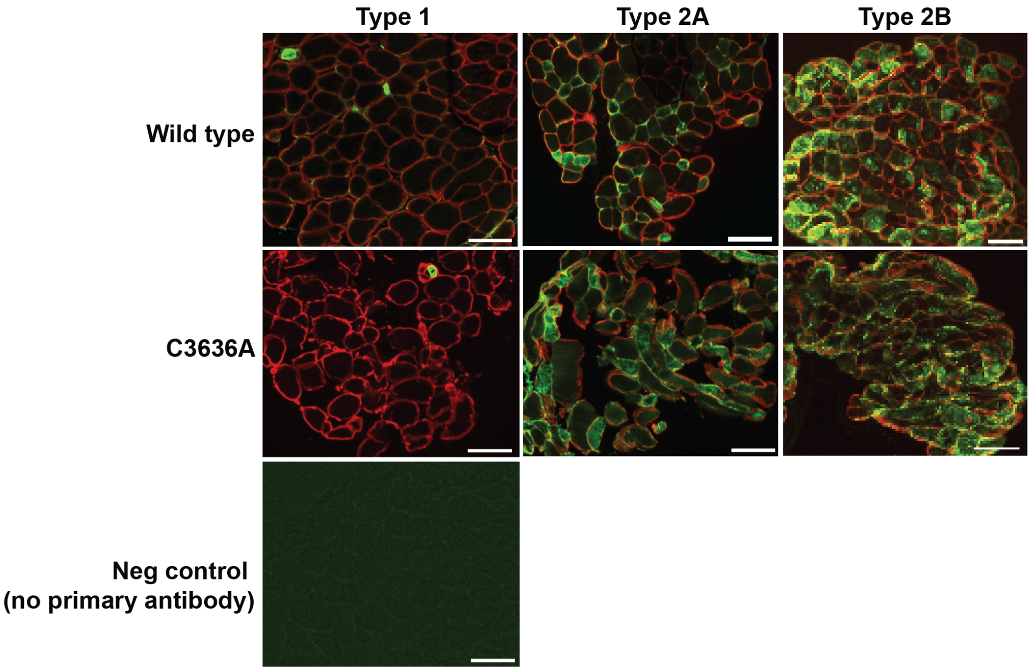

Fig. S6. Representative microscopy images of EDL muscle cross-section samples from wild type and C3636A RyR1 mice (Related to Figure 2). Muscles were stained by immunohistochemistry with antibodies specific for laminin (membrane marker; red), and muscle fiber myosin heavy chain (MHC) isoforms for Type 1 (slow twitch), Type 2A (fast twitch; oxidative) or Type 2B (fast twitch; glycolytic) fibers in green, as indicated. WT and C3636A mice were age-matched; n≥3 for each staining. All antibodies were from the University of Iowa Developmental Studies Hybridoma Bank (DSHB); Type 1: BA-F8; Type 2A: SC-71; Type 2B: BF-F3. Negative control = no primary antibody, only secondary antibody. Scale bar = 100μm.

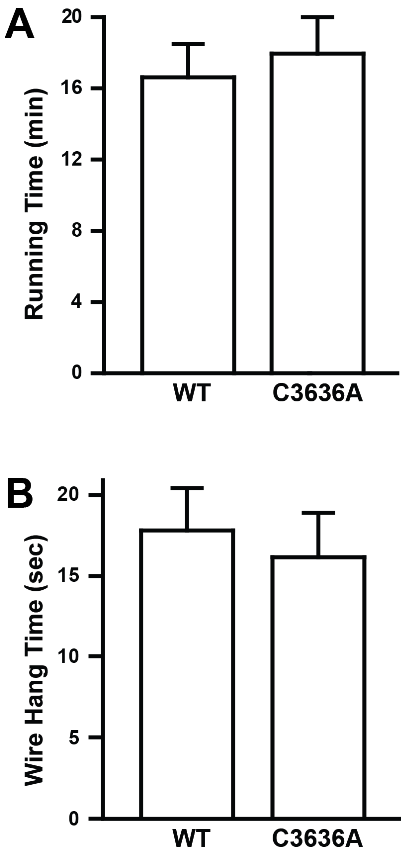

Fig S7. Muscle function in vivo (Related to Figure 3). Running time (A) and wire hang time (B) in WT and C3636A mice; n=4 mice per group.

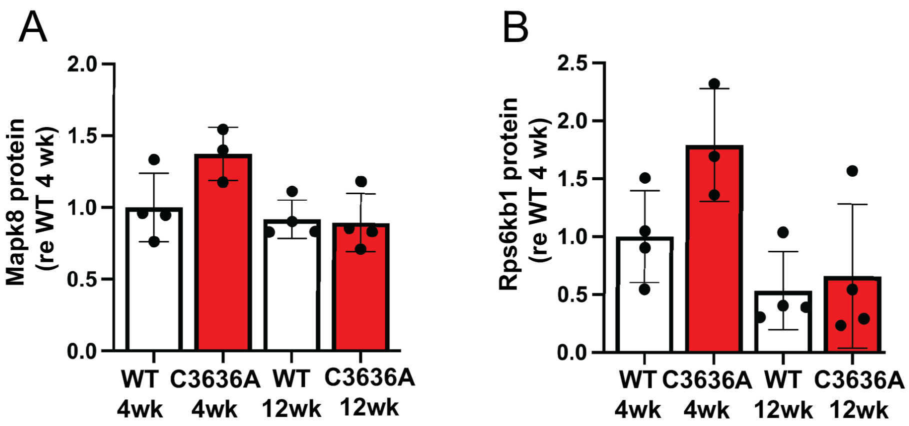

Fig S8. Expression of Mapk8 and Rps6kb1 protein in 4- and 12-week-old WT and C3636A mice (Related to Figure 4). Protein expression of Mapk8 (JNK1) (A) and Rps6kb1 (ribosomal protein S6 kinase beta-1; S6K1) (B) in hind-limb muscle isolated from WT and C3636A mice; n=3-4 mice per group.
